# Novel dual glucagon-like peptide-1/ glucose-dependent insulinotropic polypeptide receptor agonist attenuates diabetes and myocardial injury through inhibiting hyperglycemia, inflammation and oxidative stress in rodent animals

**DOI:** 10.1080/21655979.2022.2051859

**Published:** 2022-04-06

**Authors:** Ying Wang, Fei Cai, Gang Li, Yong Tao

**Affiliations:** a Department of Cardiology, Nantong Third People’s Hospital and the Third People’s Hospital Affiliated to Nantong University, Nantong, People’s Republic of China; b Department of Intensive Care Unit, Tumor Hospital Affiliated to Nantong University, Nantong Tumor Hospital, Nantong, People’s Republic of China

**Keywords:** Dual GLP-1/GIP receptor agonist, diabetic cardiomyopathy, apoptosis, oxidative stress, inflammation, mouse model

## Abstract

This study was aimed to evaluate the therapeutic effects and potent mechanisms of a novel GLP-1/GIP dual agonist on hyperglycemia and myocardial injury in diabetic mice. Novel dual-receptor agonists were designed and then evaluated via *in vitro* receptor activation assays. Acute hypoglycemic effects were assessed in diabetic mice induced by intraperitoneal injection of streptozotocin. Chronic effects of dual-receptor agonists on diabetes as well as diabetic cardiomyopathy were investigated in DCM model mice. Effects of the *in vitro* coculture of dual-receptor agonists with or without signaling pathway inhibitors on the cell viability and apoptosis of primary cardiomyocytes under a high-glucose state were assessed via MTT and western blotting methods to investigate the probable mechanism. AP5 exhibited balanced activities of dual-receptor activation *in vitro* and improved hypoglycemic ability in diabetic mice. Moreover, chronic treatment of AP5 achieved the prominently improved efficacy in reversing the deteriorative diabetic disorders and reducing the myocardial injury markers in DCM mice. Moreover, AP5 also inhibited the apoptosis and improved the survival rate of primary cardiomyocytes under a high-glucose state via increasing the expression levels of antiapoptotic proteins and inhibiting the release of apoptotic proteins, respectively, as well as activating the AMPK/PI3K/Akt signaling pathway. In conclusion, the dual GLP-1/GIP receptor agonist, AP5, can effectively improve diabetic symptoms and exert therapeutic effects on DCM via activating the AMPK/PI3K/Akt pathway, reducing the ROS production, oxidative stress and inflammatory markers in the rodent DCM model.**Abbreviation:** Diabetes mellitus, DM; diabetic cardiomyopathy, DCM; streptozotocin, STZ; glucagon-like peptide-1, GLP-1; malondialdehyde, MDA; glucose-dependent insulinotropic polypeptide, GIP; creatine kinase, CK; diabetic cardiomyopathy, DCM; serum superoxide dismutase; SOD; total superoxide disumutase, T-SOD; Methyl Thiazolyl Tetrazolium, MTT; lactate dehydrogenase; LDH; Adenosine Monophosphate-Activated Protein Kinase, AMPK; Dulbecco’s modified Eagle medium, DMEM; Fetal Bovine Serum, FBS; Reactive Oxygen Species, ROS; Glyceraldehyde-phosphate dehydrogenase, GAPDH; Surface Plasmon Resonance, SPR; Ethylene Diamine Tetraacetic Acid, EDTA; Interleukin-1β, IL-1β; Phosphoinositol 3-kinase, PI3K; Tumor necrosis factor, TNF-α; Renin-angiotensin-aldosterone system, RAAS; Glucose transporter, GLUT; Dipeptidyl peptidase-IV, DPP-IV; oxygen free radicals, OFR;

## Graphical abstract

**Figure:**
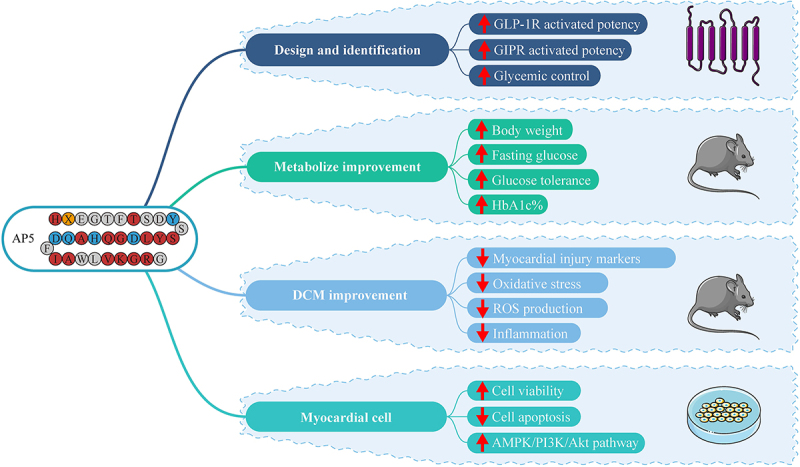

## 1. Introduction

Diabetes mellitus is a chronic endocrine disorder and metabolic disorder caused by insufficient insulin secretion, functional defects, and insulin resistance due to lifestyle changes or genetic factors [1,2]. Cardiovascular disease is one of the serious complications of diabetes, accounting for 60 to 80% of diabetes-related deaths [3,4]. Atherosclerosis is the main cause of heart failure in diabetic patients, followed by myocardial cell contractile dysfunction, known as DCM [5].

DCM first presents with myocardial fibrosis, dysfunctional and remodeled cardiomyocytes, after which diastolic and systolic dysfunction may occur, ultimately leading to heart failure [3,6]. Many reports have proposed that the pathophysiological changes of DCM include decreased AMP-activated protein kinase, abnormal peroxidase-activated receptor, O-linked N-acetylglucosamine changes, autophagy and phosphatidylinositol 3-kinase pathway abnormalities [7,8]. Blood glucose level is the result of mutual regulation of body organs, and different circulating factors including hormones, cytokines, chemokines and growth factors are known to have important information transmission effects between organs [9].

GLP-1 is a kind of incretin secreted by intestinal L cells [10]. It can promote the secretion of insulin by islet p cells after binding to GLP-1 receptor and then play a biological role in lowering blood glucose [11]. In recent years, more and more studies have confirmed that GLP-1 signaling pathway activation not only has a hypoglycemic effect but also has a cytoprotective effect [12]. It can reduce myocardial cell and endothelial cell injury induced by high glucose [13].

Similar to GLP-1, a 42-amino acid glucose-dependent insulinotropic polypeptide (GIP) has insulinotropic effects and lowers hyperglycemia [14]. In summary, both of them can promote insulin secretion, thereby upregulating the sensitivity of the insulin signal transduction pathway, so that they control blood glucose and exert the biological effect of insulin, which is one of its effects; in addition, with the continuous understanding of incretin and related drugs, it has been found that it has a wide range of physiological effects, not only a good control effect on blood glucose but also a protective effect on various tissues such as heart, kidney, and nervous system [^14–16^]. GIPR and GLP-1 R were expressed in heart, kidney and brain tissues [14]. After intraperitoneal administration into the body, GIP and GLP-1 analogs can cross the blood–brain barrier and enter the brain tissue [17,18]. After binding to the corresponding receptors, they activate a series of intracellular downstream signaling molecules to exert cytoprotective effects on the heart and other organs, with little effect on blood glucose fluctuations [14].

As mentioned above, the advantages of these two substances have made them widely used in the treatment of diabetic complications and mechanism research. Nevertheless, it is still not clear whether our newly designed GLP-1/GIP dual-receptor agonist holds protective effects on myocardial injury induced by hyperglycemia and its probable mechanisms. In this study, we hypothesized that our newly designed GLP-1/GIP dual agonist (AP5) may trigger beneficial effects on diabetic cardiomyopathy in diabetic mice. This agonist shares significant structural homology with the native GLP-1 and GIP. Furthermore, we evaluate the protective effect and the underlying mechanisms of AP5 on diabetic cardiomyopathy in rodent animals.

## 2. Methods and materials

### 2.1 Materials

All dual-receptor agonist candidates were synthesized by China Peptides (Shanghai, China) using Fmoc chemistry, and all purities were > 95%. Streptozotocin (STZ) was purchased from Sigma, USA; blood glucose test strips and supporting blood glucose meters were purchased from Roche, USA. FBS, penicillin/streptomycin and DMEM were obtained from Life Technologies, Carlsbad. Trypsin, MTT kit, ROS and apoptosis detection kits were bought from Hangzhou Biyuntian Company. Anti-BCL2, BAX, p-PIK3, cleaved Caspase 3, p-AMPK, p-Akt and β-actin were obtained from Abcam, USA.

### 2.2 In vitro *evaluation assays*

CHO cells stably expressing GIPR and GLP-1 R were used to assess *in vitro* potency of different dual-receptor agonists via measuring the expression of luciferase. The values of the EC_50_ were calculated by using Graphpad prism 6 (San Diego, USA).

### 2.3 Extraction and culture of primary cardiomyocytes

The newborn SD rats (1- to 3-day-old) were selected and fully soaked and disinfected in 75% alcohol solution. The thoracic cavity was cut open to remove the heart, and residual blood was washed off in PBS solution. The removed heart was fully cut into pieces and digested with type I collagenase at 37°C 6 times, 5 min each time. The digestive juice was centrifuged, resuspended and plated, and the solution was changed 48 hours later. The control group (CON group) was given the culture medium of 5 mmol/L glucose + 10% FBS and was cultured for 48 h, while the diabetic cell model group (HG group) was given culture medium containing 33 mmol/L glucose + 10% FBS for 48 h. Cells were assigned to the control group (no drug treatment), LPS group and dual-receptor agonist group at final concentrations of 1, 2 and 5 mmol/L for 24 h. Survival rates of primary cardiomyocytes were assessed via MTT methods according to the previously published procedures [19].

### 2.4 ROS content and cell apoptosis measurement

Cells were trypsinized with EDTA-free trypsin followed by centrifugation at 300 g for 6 min at 4°C. After removing the previous phase of centrifugation, freshly prepared binding buffer was added to resuspend the centrifuged cells to a final concentration of 10^6^ cells/mL. PBS and cells (400 μL: 100 μL) were mixed at a ratio of 4:1 in a flow cytometry tube, followed by the addition of 10 μL of PI and 5 μL of Annexin V-Alexa Fluor 488, and gently mixed and placed at room temperature in the dark for 15 min, followed by the detection of apoptosis using flow cytometry.

At the experimental end, the DCFH-DA was diluted with the mediums to the final concentration of 10 μmol/L. Then, the cell mediums were removed, and 1 mL of DCFH-DA working solution was added and then incubated at 37°C for half an hour. Cells were washed with serum-free cell culture medium, and excess DCFH was completely removed; finally, cellular ROS content was detected by flow cytometry.

### 2.5 Establishment of diabetic model mice and experimental design

Forty SPF grade wild-type C57BL mice (male, 8-week-old, weighing 20–22 g) were purchased from the Changzhou Cavens Model Animal Center. These mice were intraperitoneally injected with STZ at a dose of 60 mg/kg for 5 days, and blood glucose was measured 1 week after the injection; mice with fasting blood glucose greater than 200 mg/dL were a successful diabetic mouse model. General feeding was continued for 12 weeks to induce diabetic cardiomyopathy, and all mice had free access to food and water. All the experimental animal studies in the current study were performed according to the guideline of Experimental Animal Ethics Committee and approved by the Laboratory Animal Center of Nantong University with the code of NTCLA-IACUC-200702021.

During the experimental period, the above model mice were randomly divided into five groups: mice that daily received the saline, GLP-1 (10 nmol/kg), GIP (10 nmol/kg), GLP+GIP (5 + 5 nmol/kg) and AP5 (10 nmol/kg) for 8 weeks. Mice were continuously monitored weekly for fasting blood glucose and body weight, and glycated hemoglobin and glucose tolerance were measured 8 weeks later. After 8 weeks of treatment, the mice in all groups were sacrificed. Cardiac functions and body weights of all the mice were measured. Cardiac tissues or serum levels of CK, LDH, SOD, MDA and T-SOD were measured via ELISA methods.

### 2.6 Western blotting measurement

The separated tissues were washed with PBS 3 times, fully ground and operated at 4°C for the whole process. Proteins were extracted by adding the lysate RIPA, and the loading volume was adjusted according to the protein quantification results. SDS-PAGE protein electrophoresis was performed according to the operating procedure and transferred to the NC membrane. Five percent skimmed milk powder was prepared for blocking and incubation for 1 h, TBST solution was washed three times, primary antibodies such as β-actin and sheared caspase3 as well as other targets were incubated overnight (dilution ratio 1:1000), TBST solution was washed three times, goat antirabbit IgG antibody was added dropwise for 2 h and TBST solution was washed three times. The target protein bands were detected by chemiluminescence and recorded and analyzed with the Bio-Rad imaging system, and the level of each protein expression was standardized using β-actin as an internal reference.

### 2.7. Statistical analysis

All results were analyzed using the GraphPad Prism 8.4 (San Diego, USA). Differences between groups were determined using one-way ANOVA. P-values of 0.05 or less were considered significant.

## 3. Results

This study was aimed to determine the therapeutic effects and potent mechanisms of a novel GLP-1/GIP dual agonist on hyperglycemia and myocardial injury in diabetic mice. Novel dual-receptor agonists were designed and then evaluated via *in vitro* receptor activation assay. Acute hypoglycemic effects were assessed in the diabetic mice induced by intraperitoneal injection of streptozotocin. Chronic effects of the dual-receptor agonist on diabetes as well as diabetic cardiomyopathy were investigated in the DCM model mice. Effects of *in vitro* coculture of the dual-receptor agonist with or without signaling pathway inhibitors on the cell viability and apoptosis primary cardiomyocytes under a high-glucose state were assessed via the MTT and the western blot method to investigate the probable mechanism.

### 3.1 Design, preparation and identification of dual-receptor agonists

**Figure 1.**
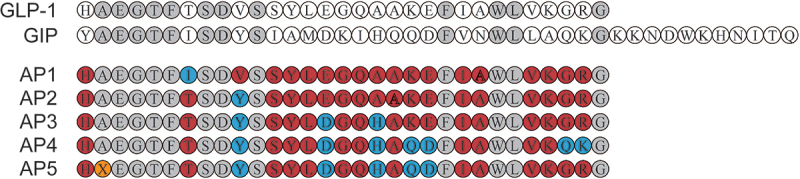
Design and schematic structures of therapeutic peptides.

**Figure 2.**
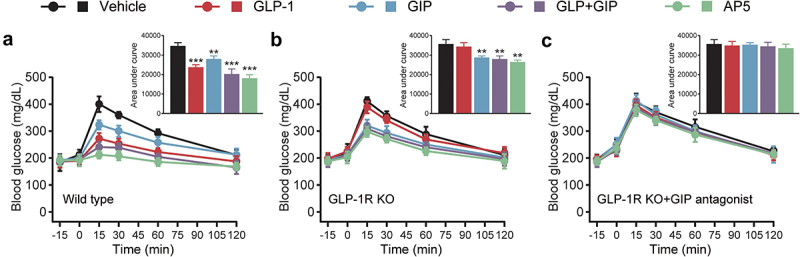
Effects of AP5 treatment on the glucose tolerance in wild-type and receptor-deficient mice. The glucose level and AUC of the (a) wild-type mice, (b) GLP-1 R KO mice and (c) GLP-1 R KO mice that received the GIP antagonist. *P < 0.05, **P < 0.02, ***P < 0.001 *vs*. saline-treated model group. Data are presented as the means ± SD (n = 6 each group).

Based on the high sequence similarity of GLP-1 and GIP, we incorporate residues from the GIP sequence other than conserved amino acids (the residues highlighted in light gray) on the basis of the GLP-1 sequence (Figure 1). In particular, Ala^8^ was replaced by aminoisobutyric acid (X) to resist degradation by DPP-IV. The potency of each peptide was evaluated in CHO cells expressing either GLP-1 R or GIPR, and the results are shown in Table 1. A significant decrease in potency of GLP-1 R was observed, while Thr^7^ in AP1 was replaced with Ile^7^, indicating that Thr^7^ is required for the activation of native GLP-1 R activity. In addition, substitution of Val^10^ into Tyr^10^ dramatically improved the activated potency of AP2 for GIPR with no apparent effect on GLP-1 R activation potency. Subsequently, we introduced Asp^15^ and His^18^ into AP3, which further improved the activated potency against GIPR. In contrast, substitution of Gln^29^ and Lys^30^ resulted in a significant decrease of GLP-1 R-activated potency in AP4, while the substitution of Gln^20^ and Asp^21^ significantly improved the activated potency of AP5 for GIPR. Therefore, AP5 was selected as a dual agonist candidate for further evaluation. The balanced *in vitro* potency of the dual agonist translated into *in vivo* activity was confirmed by comparing the acute effects of AP5 on glucose tolerance in wild-type db/db mice or those with silencing GLP-1 R signaling and/or GIPR signaling. As shown in Figure 2, in wild-type db/db mice, AP5 exerted enhanced improvement in glucose tolerance than GLP-1 R and GIPR monoagonist alone as well as combination. After GLP-1 R gene silencing, GLP-1 failed to recapitulate the improvement in glucose tolerance of the db/db mice, while AP5 still significantly improved glucose tolerance, indicating that AP5 also exhibits the GIP activity *in vivo* and also exhibits potent effects on improving glycemic control. Furthermore, AP5 failed to reproduce the similar improvement on glucose tolerance in GLP-1 R KO mice pretreated with GIPR antagonists. The above results together indicate that AP5 holds promising *in vivo* activity against both GLP-1 R and GIPR, which, in turn, achieves good acute glycemic control.

**Table 1.** Receptor activation of AP peptides for GLP-1 R and GIPR. Data are presented as the means ± SD (n = 3 each group)

Peptides	GLP-1 R EC_50_ (nM)	GIPR EC_50_ (nM)
GLP-1	0.031 ± 0.005	1625.105 ± 251.322
GIP	1587.555 ± 196.075	18.104 ± 2.201
AP1	1675.032 ± 356.434	1485.008 ± 256.018
AP2	0.028 ± 0.003	523.412 ± 57.036
AP3	0.044 ± 0.010	152.353 ± 7.478
AP4	9.072 ± 1.458	0.122 ± 0.019
AP5	0.085 ± 0.014	0.154 ± 0.008

### 3.2 Chronic treatment of AP5 improved metabolic indices associated with diabetes in DCM mice

**Figure 3.**
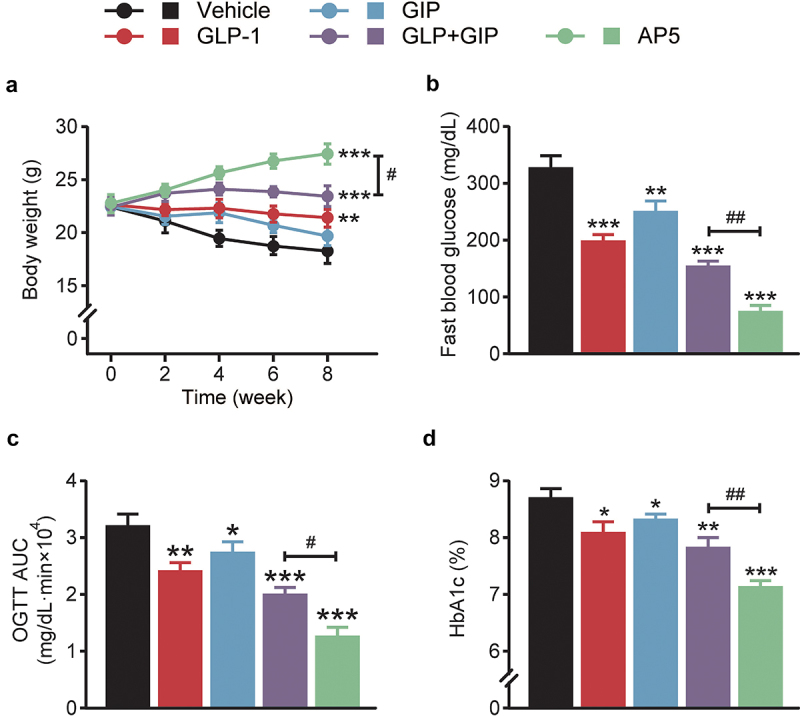
Chronic effects of AP5 treatment on the diabetic symptoms of the diabetic mice. (a) Body weight, (b) fasting blood glucose level, (c) glucose tolerance and (d) HbA1c (%). *P < 0.05, **P < 0.02, ***P < 0.001 *vs*. saline-treated model group. ^#^*P* < 0.05, ^##^*P* < 0.02 *vs*. GLP-1+ GIP group. Data are presented as the means ± SD (n = 6 each group).

To evaluate the antidiabetic effects, LM06 was subcutaneously administered once a day for consecutive 8 weeks in the DCM model. Chronic treatment of AP5 exhibited obvious body weight lowering effects on the model mice, while those of saline-treated ones continued to increase during the entire experimental period (Figure 3a). Interestingly, chronic treatment of AP5 exhibited enhanced improvement in the fasting blood glucose level of DCM mice compared with those of saline or other ones treated DCM mice (Figure 3b). OGTT was conducted after 8-week treatment, and the results showed that AP5 significantly decreased the AUC_0-120min_ value (Figure 3c). Not only that, chronic treatment of AP5 also induces a significant decrease in the %HbA1c value, which is also significantly lower than any other group (Figure 3d).

### 3.3 Dual-receptor agonist ameliorates myocardial injury markers in DCM mice

**Figure 4.**
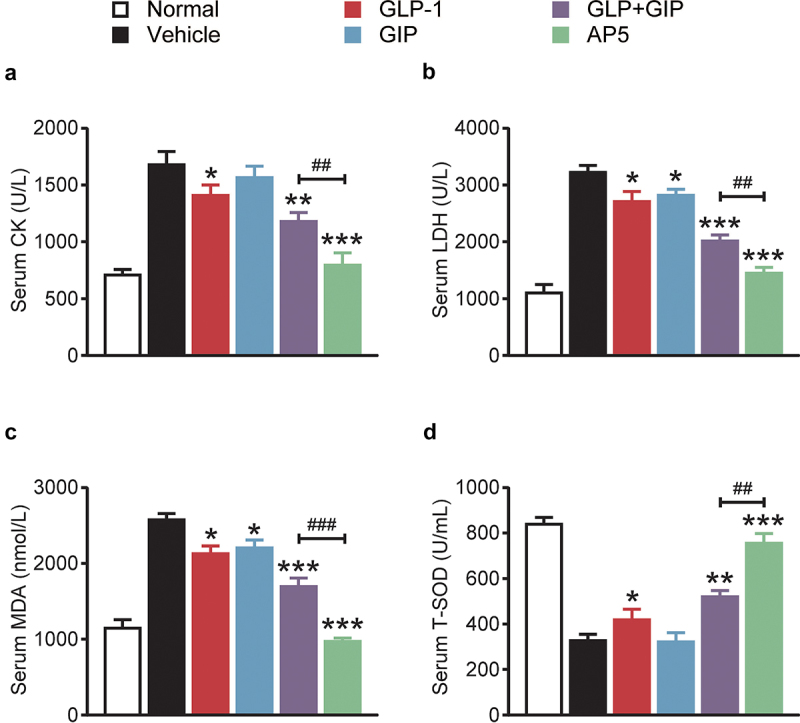
Chronic therapeutic efficacy of AP5 on serum myocardial markers, myocardial injury and the accumulation of lipids in DCM mice. The serum level of (a) CK, (b) LDH, (c) MDA and (d) T-SOD. *P < 0.05, **P < 0.02, ***P < 0.001 *vs*. saline-treated model group. ^#^*P* < 0.05, ^##^*P* < 0.02, ^###^*P* < 0.001 *vs*. GLP-1+ GIP group. Data are presented as the means ± SD (n = 6 each group).

As shown in Figure 4, the serum levels of LDH and CK in DCM mice in the control group were significantly higher than those in the normal mouse group, suggesting the occurrence of cardiac injury in mice under high-glucose conditions. After 8 weeks of treatment, the serum CK and LDH levels in the dual-receptor agonist group were significantly lower than those in the model control group and were also significantly lower than those in GLP-1 and GIP alone or combination, suggesting that dual-receptor agonist administration reduced the cardiac injury triggered by high glucose and present the advantage of fusion molecules. Moreover, the MDA content in the serum of mice in the model control group was also significantly higher than that in the normal mouse group, suggesting that there was oxidative stress in the myocardial tissues of mice under a long-term high-glucose state, and the results of combined pathological analysis showed that there was more severe myocardial injury. Consistent with the trend of serum levels of CK and LDH, continuous administration of AP5 significantly reversed the upregulation of serum MDA levels and was significantly superior to GLP-1 and GIP alone or combination (all *p* < 0.05), suggesting that continuous administration of AP5 can reduce oxidative stress response and free radical damage to cells in myocardial tissue. Similarly, changes in T-SOD activity also indicate that AP5 exhibited significant antioxidant effects, increasing the serum SOD levels and reducing the OFR in cardiac myocytes.

### 3.4 AP5 inhibits ROS production and inflammation-related proteins in diabetic model mice

**Figure 5.**
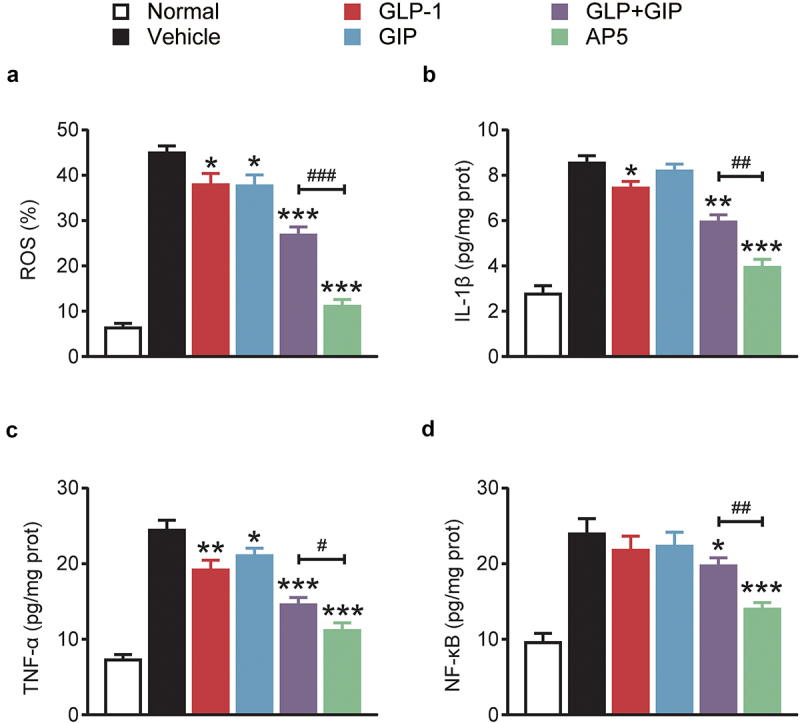
Chronic treatment of AP5 prevents the hyperglycemia, cardiac inflammations and oxidative stress in diabetic mice. The (a) ROS% and the expression levels of (b) IL-1β, (c) TNF-α and (d) NF-κB. *P < 0.05, **P < 0.02, ***P < 0.001 *vs*. saline-treated model group. ^#^*P* < 0.05, ^##^*P* < 0.02, ^###^*P* < 0.001 *vs*. GLP-1+ GIP group. Data are presented as the means ± SD (n = 6 each group).

The expression of ROS in cardiomyocytes was further investigated. As shown by the results of Figure 5, the production of ROS in cardiomyocytes of the model control group was significantly higher than that of the normal control group (*p* < 0.01), suggesting the generation of oxidative stress. After long-term administration, AP5 significantly decreased ROS production in cardiomyocytes, and there were significant differences in GIP and GLP-1 alone or combination compared to the model control group (all *p* < 0.01). We further evaluated the expression of inflammatory markers, including IL-1β, TNF-α and NF-κB, in the myocardial tissues by means of ELISA (Figure 5). The results showed that the three inflammation-related proteins in the myocardial tissue of the model control group were significantly increased compared with those of the normal mice, indicating that the myocardial tissue had an inflammatory response. After 8 weeks of long-term administration, the level of IL-1β, TNF-α and NF-κB in the dual-receptor agonist GLP-1 group was significantly lower than that in the placebo group (all *p* < 0.05). Interestingly, the ameliorative effect of AP5 exhibits significant advantages over GLP-1 and GIP alone or in combination, suggesting the ameliorative advantage of fusion molecules. The above results indicate that AP5 can inhibit the inflammatory response via significantly downregulating the release of proinflammatory factors using its own advantages of fusion molecules, thereby protecting cardiac tissues from the inflammatory damage.

### 3.5 AP5 improves the cell viability and apoptosis of high-glucose-treated primary cardiomyocytes via activating the AMPK/PI3K/Akt pathway

**Figure 6.**
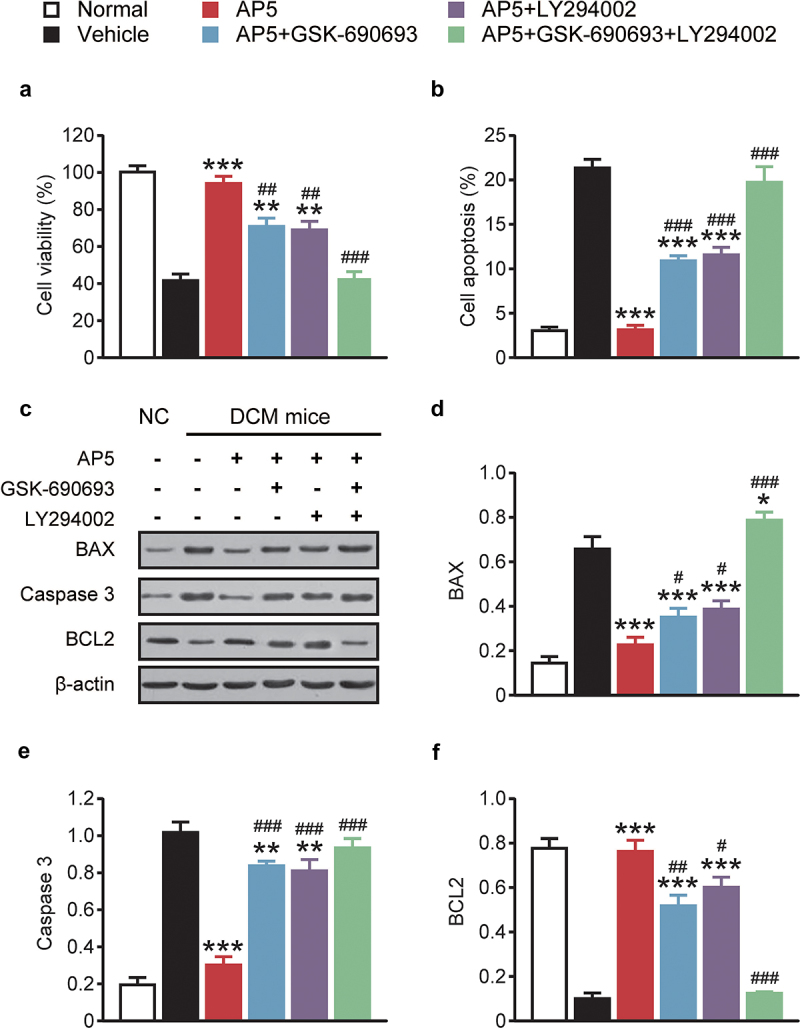
Chronic effects of AP5 treatment on (a) viability, (b) apoptosis and (c) and (d) apoptosis-related factors of primary cardiomyocytes. *P < 0.05, **P < 0.02, ***P < 0.001 *vs*. saline-treated model group. ^#^*P* < 0.05, ^##^*P* < 0.02, ^###^*P* < 0.001 *vs*. AP5 alone-treated model group. Results are shown as means ± SD (n = 3 each group).

MTT assay was used to assess the protective effect of AP5 on the viability of primary cardiomyocytes. As shown in Figure 6a, high-glucose treatment significantly reduced the viability of primary cardiomyocytes compared with normal controls (*p* < 0.05). In contrast, the viability of primary cardiomyocytes in the dual-agonist coincubation group was significantly higher than that in the high-glucose alone group (all *p* < 0.05). Moreover, we further measured the fraction of late apoptotic cells, and compared to the normal control group, the apoptotic percentage of primary cardiomyocyte was significantly increased in the single-high-glucose group and the apoptosis rate of primary cardiomyocytes in the dual-receptor agonist incubation group was also significantly lower than that in the single-high-glucose incubation group, with statistically significant differences (all *p* < 0.05). The effect of AP5 on the expression of apoptosis-related proteins in primary cardiomyocytes was further evaluated. As shown in Figures 6D-F, the expressions of Caspase 3 and BAX were obviously upregulated in mono-high-glucose incubation compared with the normal control group (both *p* < 0.05). Significantly downregulated BAX and Caspase 3 protein levels were observed in cells incubated with the dual agonist, compared with the model control group (*p* < 0.05). Moreover, the expression of BCL2 protein in the dual-receptor agonist group showed an upregulation trend, and the statistical difference with the other coincubation groups was similar to the above two markers. Interestingly, our improvement was substantially reduced after adding AMPK and PI3K inhibitors GSK-690693 (AMPK-specific inhibitor) and LY294002 (PI3K-specific inhibitor), respectively, and the improvement was almost undetectable after coincubation of the two inhibitors, suggesting that the AMPK/PI3K/Akt signaling pathway may be associated with AP5 amelioration of myocardial injury in diabetic models.

**Figure 7.**
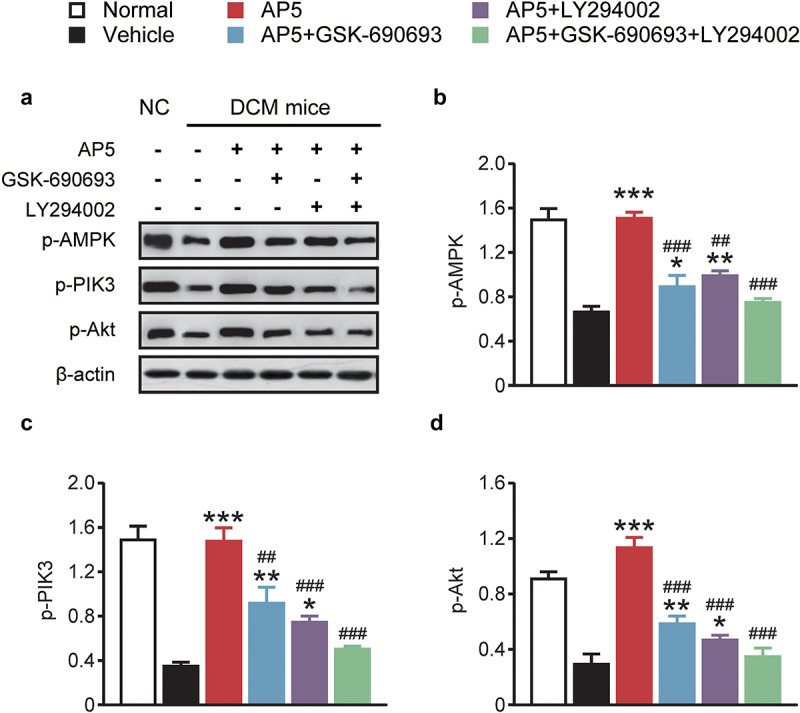
Chronic effects of AP5 treatment on the AMPK/PI3K/Akt signaling pathway-related proteins. (a) Western blotting image and the analysis of the expressions of (b) p-AMPK, (c) p-PIK3 and (d) p-Akt. *P < 0.05, **P < 0.02, ***P < 0.001 *vs*. saline-treated model group. ^#^*P* < 0.05, ^##^*P* < 0.02, ^###^*P* < 0.001 *vs*. AP5 alone-treated model group. Results are shown as means ± SD (n = 3 each group).

We therefore further investigated the expression of AMPK/PI3K/Akt signaling pathway-related proteins. As shown in Figure 7, incubation with the single-high-glucose, dual-receptor agonist group exhibited significantly increased expression levels of p-AMPK, p-PI3K and p-Akt (*p <* 0.05). Both inhibitors alone or in combination significantly decreased the expression of related proteins, which remained consistent with the above trends in inflammatory factors and apoptosis-related proteins. The above results together demonstrate that AP5 improves the viability and inhibits the apoptosis of high-glucose-treated primary cardiomyocytes via activating the AMPK/PI3K/Akt pathway.

## 4. Discussion

It has been reported that diabetes can reduce the corresponding GLUT1 and GLUT4, then reduce the activity of the glucose utilization and finally cause abnormal glucose metabolism [20,21]. It has been shown that the cardiac dysfunction of db/db mice overexpressing hGLUT4 can be reversed, thus also illustrating that abnormal glucose metabolism in the heart can lead to systolic dysfunction in diabetic hearts [22]. In the current study, it has been shown that our dual GLP-1/GIP receptor agonist can effectively improve hyperglycemia, including fasting blood glucose, glucose tolerance and glycosylated hemoglobin, in diabetic mice.

Recent findings have shown that mitochondrial function impairment plays a very important role in the pathogenesis of DCM [23]. Myocardial tissue mitochondria in DCM patients often have different degrees of damage, and functionally injured mitochondria cause impaired energy supply to cardiomyocytes, while exacerbating the level of oxidative stress and apoptosis in cardiomyocytes, ultimately causing cardiac function damage [3,24]. Recent reports have shown that hyperglycemia can induce the formation of short and small mitochondria or mitochondrial fragments in H9C2 cardiomyocytes or newborn rat cardiomyocytes [25]. Insufficient glycolysis reduces the level of oxidative phosphorylation and reduces mitochondrial ATP synthesis [26]. Hyperglycemia can not only cause damage to cardiomyocytes, fibroblasts and endothelial cells but also cause increased ROS, decreased ATP synthesis and mitochondrial dysfunction and induce myocardial apoptosis [25]. In the pathophysiological mechanism of diabetes, abnormal glucose metabolism and significantly limited glucose utilization increase fat metabolism, which further leads to an increase in ROS, while increased fat metabolism affects the storage of sarcoplasmic reticulum calcium pump calcium and impairs myocardial contractile function [7]. In experimental animal models, multiple ROS scavengers or antioxidants are able to reduce cardiomyocyte death and attenuate diabetic cardiac injury [27]. Myocardial tissue undergoes the lipid peroxidation to induce the production of MDA, which subsequently damages the cardiomyocyte and then leads to cardiac insufficiency [4]. SOD, which is one of the important antioxidant enzymes in vivo, holds the functions of scavenging free radicals and reduces the oxidative stress [4]. In the present study, chronic administration of the dual-receptor agonist effectively increased T-SOD activity in vivo, indicating a reduced oxidative stress response in cardiac tissues. Significantly, a similar ameliorative effect was also shown for the MDA production.

In addition, the mitochondrial apoptosis pathway is one of the most widely studied mechanisms of myocardial apoptosis, and excessive activation of the mitochondrial apoptosis pathway is a pathological link that plays an important role in the process of myocardial cell injury [28]. BAX and BCL2 are important molecules regulating apoptosis in the mitochondrial pathway; hypoxia as well as hypoxia reoxygenation can increase the expression of BAX, translocate BAX to the mitochondrial surface and increase the permeability of mitochondrial membranes to cytochrome c and cytochrome c entering the cytoplasm from mitochondria can initiate the caspase cascade and ultimately activate caspase-3 to perform apoptosis [29]. Bc1-2 is able to form a heterodimer with BAX and impede the translocation of BAX to the mitochondrial surface, which, in turn, inhibits apoptosis of the mitochondrial pathway [29]. In this study, we also detected the levels of oxidative stress and apoptosis in diabetic myocardium at two levels: animal and cell, and found that the levels of oxidative stress and apoptosis in mitochondria were significantly higher in myocardial tissue of STZ-induced diabetic mice and high-glucose-induced diabetic primary cardiomyocytes, indicating that mitochondrial oxidative stress and apoptosis are significant characteristics of DCM and inhibition of mitochondrial oxidative stress and apoptosis may be an important strategy for the treatment of DCM. The results of this study also showed that the protein expression of BAX and caspase-3 in cardiomyocytes of the model group was significantly higher than that of the control group, the protein expression of BCL2 was significantly lower than that of the control group, the protein expression of BAX and caspase-3 in cardiomyocytes of the dual-receptor agonist treatment group was significantly lower than that of the model control group and the protein expression of Bc1-2 was significantly higher than that of the model control group, suggesting a significant improvement effect on myocardial mitochondrial pathway apoptosis.

Diabetic cardiomyopathy is associated with a variety of signaling pathways, such as AMPK, Nrf2-ARE signaling pathway, Wnt/β-catenin signaling pathway, NF-κB signaling pathway, TGF-β1/Smads signaling pathway, PKC signaling pathway and PI3K/AKT signaling pathway [30]. It has been reported that curcumin derivative C66, an inhibitor of JNK phosphorylation, can reduce the high-glucose-activated JNK/NF-κB pathway in diabetic cardiomyopathy mice, thereby reducing the inflammatory response and apoptosis process and delaying the process of diabetic cardiomyopathy [31]. In the stage of diabetic cardiomyopathy, a variety of mechanisms and signaling pathways will promote pathological changes in cardiomyocytes, ultimately leading to arrhythmia and heart failure [30]. The pathogenesis of diabetic cardiomyopathy is complex and not yet fully understood. The AMPK/PI3K/Akt signaling pathway is an important pathway regulating biological processes such as apoptosis, survival and proliferation [32]. AMPK is a kind of energy sensor, which can regulate the balance of the cellular energy [33]. In addition, the AMPK is also considered as the regulator of cardiac energy metabolism, and the phosphorylated AMPK, which is involved in the metabolism of cardiomyocyte energy, can reduce cardiomyocytes injuries [33]. PI3K is able to act on Akt and phosphorylate it also after activation in the form of phosphorylation [34]. Phosphorylated Akt can regulate the expression of target genes, and promote cell proliferation and inhibit apoptosis [34]. In this study, the expression levels of p-AMPK, p-PI3K and p-Akt in the cardiomyocytes of the model group were significantly decreased, while the expression levels of the three phosphorylation markers in the cardiomyocytes of the model group were significantly increased, suggesting that dual-receptor agonists can activate the AMPK/PI3K/Akt signaling pathway, and the signaling pathway inhibitors GSK-690693 (AMPK-specific inhibitor) and LY294002 (PI3K-specific inhibitor) were used to treat the cardiomyocytes with dual-receptor agonists. The results suggest that dual-receptor agonists do prevent the cardiomyocyte injury through the AMPK/PI3K/Akt signaling pathway.

## 5. Conclusion

In summary, our newly designed dual GLP-1/GIP receptor agonist, AP5, can effectively improve the diabetic symptoms and exert protective effects on diabetic cardiomyopathy via inducing the activation of the AMPK/PI3K/AKT signaling pathway, reducing ROS production, oxidative stress and proinflammatory factors in the DCM mice.
